# Effects of Hypoglycemia on Cardiovascular Function in Patients with Diabetes

**DOI:** 10.3390/ijms24119357

**Published:** 2023-05-27

**Authors:** Maria A. Christou, Panagiota A. Christou, Christos Kyriakopoulos, Georgios A. Christou, Stelios Tigas

**Affiliations:** 1 Department of Endocrinology, University of Ioannina Faculty of Medicine, 45500 Ioannina, Greece; mariachristou1@gmail.com (M.A.C.); panagiotaxrist@hotmail.com (P.A.C.); 2 Department of Respiratory Medicine, University of Ioannina Faculty of Medicine, 45500 Ioannina, Greece; ckyriako@yahoo.gr; 3 Laboratory of Physiology, University of Ioannina Faculty of Medicine, 45500 Ioannina, Greece; georgios.christou@yahoo.gr

**Keywords:** hypoglycemia, diabetes, cardiovascular, mechanisms, ischaemia, arrhythmias, thrombosis, inflammation, oxidative stress

## Abstract

Hypoglycemia is common in patients with type 1 and type 2 diabetes (T1D, T2D), treated with insulin or sulfonylureas, and has multiple short- and long-term clinical implications. Whether acute or recurrent, hypoglycemia significantly affects the cardiovascular system with the potential to cause cardiovascular dysfunction. Several pathophysiological mechanisms have been proposed linking hypoglycemia to increased cardiovascular risk, including hemodynamic changes, myocardial ischemia, abnormal cardiac repolarization, cardiac arrhythmias, prothrombotic and proinflammatory effects, and induction of oxidative stress. Hypoglycemia-induced changes can promote the development of endothelial dysfunction, which is an early marker of atherosclerosis. Although data from clinical trials and real-world studies suggest an association between hypoglycemia and cardiovascular events in patients with diabetes, it remains uncertain whether this association is causal. New therapeutic agents for patients with T2D do not cause hypoglycemia and have cardioprotective benefits, whereas increasing the use of new technologies, such as continuous glucose monitoring devices and insulin pumps, has the potential to reduce hypoglycemia and its adverse cardiovascular outcomes in patients with T1D.

## 1. Introduction

Hypoglycemia is a major barrier to maintaining satisfactory glycaemic control in patients with diabetes. Level 1 hypoglycemia is defined as blood glucose concentration <70 mg/dL but ≥54 mg/dL, level 2 hypoglycemia as glucose <54 mg/dL in the presence of neuroglycopenic symptoms, and level 3 hypoglycemia as a severe event characterized by altered mental and/or physical functioning that requires assistance from another person [1]. There are several factors that increase hypoglycemia risk in diabetes, such as the use of insulin or sulfonylureas, impaired renal or hepatic function, diabetes duration, frailty and old age, cognitive impairment, impaired hypoglycemia awareness, a physical or intellectual disability that impairs behavioral response to hypoglycemia, alcohol use, polypharmacy, and history of severe hypoglycemia [2].

It is difficult to accurately estimate the true frequency of hypoglycemia in patients with diabetes due to differences in the definitions used and in the characteristics of the studied populations. A patient with type 1 diabetes (T1D) experiences around two episodes of symptomatic hypoglycemia per week and at least one severe episode per year [3]. It has been reported that the mean incidence (95% confidence interval, CI) of severe hypoglycemia in patients with T1D and disease duration <5 years is 1.1 (0–2.3) episodes per person-year, whereas it increases to 3.2 (1.6–4.9) in those with diabetes duration >15 years [4]. Hypoglycemia is less common in patients with insulin-treated type 2 diabetes (T2D), with 3–25% of patients experiencing at least one severe episode annually [5]. However, the incidence of severe hypoglycemia in patients with T2D who have been treated with insulin for >5 years increases and approaches that of patients with T1D [4]. The annual prevalence of severe hypoglycemia in patients treated with sulfonylureas is 7% [4]. Continuous glucose monitoring (CGM) data suggest that asymptomatic hypoglycemia is common, with at least one episode reported in 77% and 52% of patients with T1D over a 6-day period at 70 and 54 mg/dL, respectively [6].

Hypoglycemia has multiple clinical implications [5]. It can disrupt everyday activities and may have psychological consequences in patients with diabetes (e.g., fear of hypoglycemia, negative effects on mood and emotion), adversely affecting the quality of life [7,8]. In addition, it may lead to accidents, injuries, cognitive impairment, cerebrovascular events, and even death. Hypoglycemia poses an economic burden to healthcare resources, especially for the treatment of severe episodes requiring admission to the hospital [9]. Furthermore, hypoglycemia exerts multiple adverse effects on the cardiovascular system, with the potential to cause cardiovascular dysfunction in patients with diabetes. Importantly, cardiovascular disease is the most common cause of death in T1D or T2D patients [10,11]. During recent years, data from large-scale clinical trials demonstrated that strict glycaemic control might reduce long-term cardiovascular complications, whereas, on the other hand, hypoglycemia can cause cardiovascular dysfunction and promote acute cardiovascular events [12,13]. Understanding the pathophysiological mechanisms potentially linking hypoglycemia to increased cardiovascular risk is essential to effectively guide the prevention and management of related adverse clinical outcomes. In this review, we aim to describe the cardiovascular effects of hypoglycemia in patients with diabetes.

## 2. Hormonal Responses to Hypoglycemia and Impaired Hypoglycemia Awareness in Diabetes

Several physiological responses are activated to counterbalance decreasing glucose levels and maintain glucose homeostasis. This is important to ensure that a sufficient amount of glucose is available to all tissues and primarily to the brain, which depends on glucose as its main source of energy [14,15,16]. The first defense step against hypoglycemia in healthy subjects is the inhibition of insulin secretion by pancreatic β-cells when blood glucose concentration falls below 80–85 mg/dL (Table 1). The reduction of insulin blood levels results in increased endogenous glucose production, which under normal circumstances is supplemented by oral carbohydrate intake. If blood glucose levels fall below 65–70 mg/dL, secretion of the counterregulatory hormones is activated; thus, glucagon secretion from the pancreatic α-cells is the second defense step leading to an increase in hepatic glucose production via stimulation of glycogenolysis. The third defense step consists of the sympathoadrenal response via activation of specific hypothalamic autonomic centers within the brain. This leads to the secretion of epinephrine and is associated with the development of autonomic symptoms (Table 1). Epinephrine acts by several mechanisms, including stimulation of glycogenolysis, an increase of hepatic and renal gluconeogenesis via mobilization of gluconeogenic substrates, such as lactate and amino acids, and reducing insulin secretion. In addition to the above, sufficient cortisol and growth hormone levels are also required as part of the normal counterregulatory response to hypoglycemia. Neuroglycopenic symptoms due to brain glucose deprivation become evident when the glucose falls below 50 mg/dL, whereas severe and prolonged hypoglycemia may result in serious consequences, such as coma, brain damage or even death. Importantly, the glucose thresholds depicted in Table 1 have been observed under experimental conditions in non-diabetic subjects [16]. However, the glucose counter-regulation against hypoglycemia and the onset of symptoms is compromised in patients with diabetes with the above glycaemic thresholds shifting to lower levels in response to changes in glycaemic control and exposure to extremes of blood glucose [17]. In patients with T1D or longstanding T2D, apart from the pancreatic β-cell failure, there is commonly defective glucagon secretion, as well as an impaired sympathoadrenal response during hypoglycemia [16]. 

**Figure 1:**
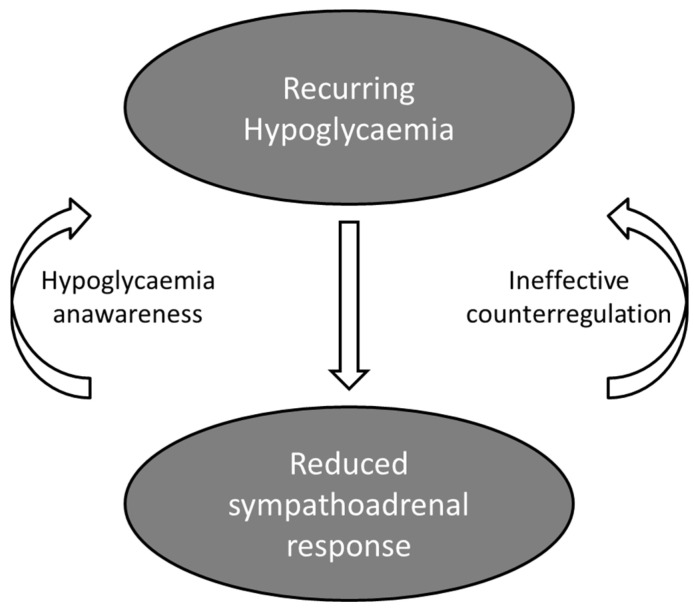
The vicious cycle of hypoglycemia-associated autonomic failure in T1D.

Impaired awareness of hypoglycemia (IAH) is common in insulin-treated patients with diabetes and correlates with the duration of insulin treatment [16]. IAH is characterized by a diminished ability to perceive the onset of acute hypoglycemia, including altered symptom profile, reduced intensity and a number of symptoms, and in some cases, failure to interpret symptoms. It does not necessarily develop into complete unawareness of hypoglycemia and may vary in severity over time, probably due to environmental influences. Patients with IAH do not experience the autonomic warning symptoms and are at risk for neuroglycopenia and severe hypoglycemia. Possible mechanisms for IAH include the following: (i) central nervous system adaptation due to chronic exposure or recurrent transient exposure to low blood glucose, (ii) central nervous system glucoregulatory failure due to counterregulatory deficiency or hypoglycemia-associated autonomic failure (HAAF), and (iii) peripheral nervous system dysfunction due to peripheral autonomic neuropathy or reduced peripheral adrenoreceptor sensitivity. The term HAAF has been proposed to describe an autonomic disorder characterized by defective glucose counter-regulation evidenced by attenuated glucagon and epinephrine responses and absent or impaired awareness of neuroglycopenic symptoms due to attenuated sympathoadrenal responses to hypoglycemia (Figure 1) [17,18]. Potential stimuli of HAAF include recent antecedent hypoglycemia, thus creating a vicious cycle of recurrent hypoglycemia, prior exercise or sleep. Importantly, HAAF is a reversible disorder pathophysiologically distinct from classic diabetic autonomic neuropathy, which is a structural disorder characterized by nerve fiber loss. 

## 3. Pathophysiologic Mechanisms Linking Hypoglycemia to Increased Cardiovascular Risk

**Figure 2:**
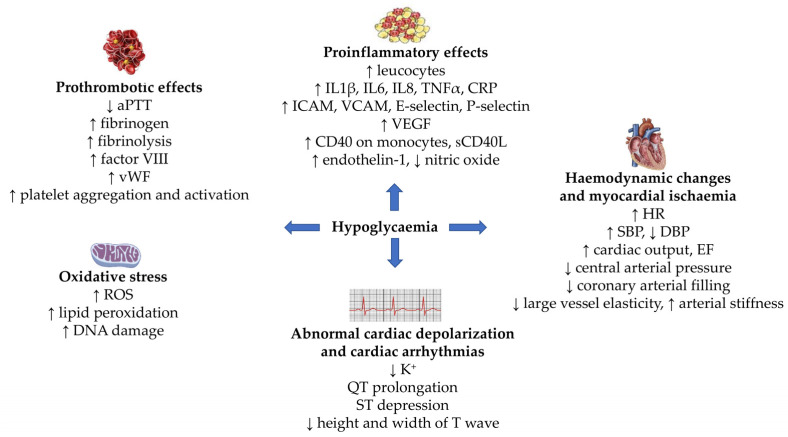
Potential pathophysiological mechanisms linking hypoglycemia to cardiovascular outcomes. Abbreviations: aPTT: activated partial thromboplastin time; CD40: a cluster of differentiation 40; CRP: C-reactive protein; DBP: diastolic blood pressure; EF: ejection fraction; HR: heart rate; ICAM: intercellular adhesion molecule; IL: interleukin; ROS: reactive oxygen species; SBP: systolic blood pressure; sCD40L: a soluble cluster of differentiation 40 ligand; TNFα: tumor necrosis factor-alpha; VCAM: vascular cell adhesion molecule; VEGF: vascular endothelial growth factor; vWF: von Willebrand factor.

Several mechanisms are implicated in the development of cardiovascular dysfunction related to hypoglycemia in patients with diabetes. These include hemodynamic changes, myocardial ischemia, abnormal cardiac repolarization, cardiac arrhythmias, thrombotic tendency, oxidative stress, and inflammation (Figure 2). Relevant evidence is derived from in vitro and in vivo studies, as well as from experimentally induced hypoglycemia studies in humans.

### 3.1. Haemodynamic Changes and Myocardial Ischemia 

Hypoglycemia activates the sympathoadrenal system causing the secretion of catecholamines that exert significant hemodynamic effects. Specifically, sympathetic system activation leads to increased heart rate, systolic blood pressure, cardiac output and ejection fraction, and decreased diastolic blood pressure [19,20]. Importantly, in patients with long-standing diabetes, the elasticity of large blood vessels and central arterial pressure decrease [21]. Progressive development of arterial stiffness leads the reflected arterial pressure wave to return earlier to the central aorta and the heart during late systole (instead of early diastole), compromising in this way coronary arterial filling and predisposing to myocardial ischemia [22].

### 3.2. Abnormal Cardiac Repolarization and Cardiac Arrhythmias

Experimental studies in rats suggest a central role for catecholamines and the sympathetic nervous system in the occurrence of cardiac arrhythmias during severe hypoglycemia. Specifically, severe lethal hypoglycemia can initially cause sinus tachycardia and QT prolongation, followed by premature atrial and ventricular contractions, first-degree atrioventricular block and ventricular tachycardia, evolving to second-degree and third-degree atrioventricular block and finally bradycardia and cardiorespiratory arrest [23]. Hypoglycemia-induced mortality in this rat model was worsened by diabetes, but recurrent hypoglycemia, potassium supplementation and β-adrenergic blockade tended to reduce sympathoadrenal activity and mortality. In another study, insulin deficiency in streptozotocin-induced diabetic rats was associated with twice the risk of third-degree atrioventricular block and death during severe hypoglycemia compared with nondiabetic rats [24]. However, exposure to recurrent hypoglycemia reduced the occurrence of third-degree atrioventricular block and mortality, implying reduced sympathoadrenal response to hypoglycemia. 

In humans, hypoglycemia can affect cardiac repolarization and electrophysiology, producing electrocardiographic changes that include ST segment depression, a decrease in height and width of the T wave, and QT interval prolongation [25,26,27]. It has been suggested that a rapid decrease in blood glucose may affect the time of onset of QT interval prolongation [28]. During prolonged periods of hypoglycemia (plasma glucose < 54 mg/dL for 80 min), progressive prolongation of the QT interval has been observed [29]. Importantly, QT prolongation increases the risk of ventricular arrhythmias [30] and is a strong predictor of cardiovascular and all-cause mortality in patients with diabetes [31,32]. Additionally, heart rate variability response to hypoglycemia is impaired in people with T2D, resulting in a higher-than-expected risk for sudden arrhythmia following mild hypoglycemic episodes [33]. Sympathoadrenal activation and catecholamine-induced hypokalemia seem to be responsible for the above arrhythmogenic changes [34]. However, frequent or recent exposure to hypoglycemia, as well as the development of cardiac autonomic neuropathy, blunts the sympathoadrenal response during hypoglycaemic episodes [18,35]. It has been hypothesized that subjects with polymorphisms of the ion channels genes which contribute to the cardiac conduction system might be vulnerable to the arrhythmogenic effects of hypoglycemia [36]. However, in susceptible patients who are at high cardiovascular risk, more than one mechanism might contribute to potentially fatal cardiac arrhythmias during hypoglycemia. Several types of cardiac arrhythmias have been associated with hypoglycemia, ranging from severe sinus bradycardia and atrial fibrillation to multiple ventricular ectopics and ventricular tachycardia [34].

The observed differences in the occurrence of cardiac arrhythmias between daytime and nocturnal hypoglycemia might be associated with insufficient counter-regulation in response to hypoglycemia during sleep [37,38]. This can result in a prolonged duration of nocturnal hypoglycemia, as well as attenuation of the sympathoadrenal activation and catecholamine secretion. Attenuated sympathoadrenal activity at night leads to compensatory activation of the parasympathetic system, thus increasing the frequency of bradycardia and the risk of ventricular arrhythmias. Hypoglycemia during the day can also increase the risk of arrhythmias, but these are less likely to be life-threatening [5].

### 3.3. Prothrombotic Effects

Hypoglycemia induces a procoagulant and prothrombotic state, including promoted platelet aggregation and activation [39]. The mechanisms underlying both processes appear to be adrenaline-mediated and inhibited by alpha-2 adrenergic blockade [22]. Platelet factor 4 and plasma β thromboglobulin increase by hypoglycemia and are thought to represent the activation of platelets in vivo [22]. Accelerated rates of thrombin generation and formation of denser fibrin clots, displaying lower liability, have also been documented in patients with diabetes in response to low blood glucose [40]. Additionally, the fibrinolytic system is activated, as evidenced by the increase in tissue plasminogen activator (tPA) and decrease in plasminogen activator inhibitor (PAI) activity [41]. Euglobulin lysis time (an indicator of plasminogen activator activity) is decreased, and fibrin plate lysis increases, also indicating increased fibrinolysis.

Activated partial thromboplastin time (aPTT) is reduced, fibrinogen and factor VIII increase and platelet count is reduced following hypoglycemia in patients with diabetes [42]. Hypoglycemia induces the release of von Willebrand Factor (vWF) from endothelial cells and platelets, and vWF, in turn, promotes platelet aggregation and prolongs the lifetime of factor VIII [41]. Hypoglycemia has acute and persistent prothrombotic effects that persist for at least seven days and are enhanced in patients with diabetes [43]. Interestingly, hypoglycemia can influence the expression of platelet-related miRNAs in patients with T2D, with a time trend paralleling the time course of platelet activation [44], suggesting that miRNAs are probably released by platelets upon activation by hypoglycemia.

### 3.4. Proinflammatory Effects

Hypoglycemia can induce an inflammatory response, including mobilization of leukocytes, an increase in cluster of differentiation 40 (CD40) expression on mononuclear cells, plasma soluble CD40 ligand (sCD40L) levels and platelet-monocyte aggregation. It can also increase the levels of different adhesion markers, such as intercellular adhesion molecule (ICAM), vascular cell adhesion molecule (VCAM), P-selectin, E-selectin, as well as tumor necrosis factor alpha (TNFα), interleukin 1β (IL1β), IL6, IL8, and vascular endothelial growth factor (VEGF) levels [45,46,47]. Hypoglycemia increases leukocyte numbers in healthy subjects and patients with T1D and normal hypoglycemia awareness but not in those with IAH [48]. Ex vivo, peripheral blood mononuclear cells and monocytes display a more robust cytokine response to microbial stimulation after hypoglycemia compared with euglycemia. This response is less pronounced in patients with T1D and IAH, indicating impaired counterregulatory hormone responses in these patients [48].

In response to hypoglycemia, the numbers of lymphocytes and monocytes, as well as blood levels of C-reactive protein and other inflammatory proteins, significantly increase and may remain elevated for seven days in patients with T2D [49]. It has been shown that a longer duration of hypoglycemia results in a greater increase in proinflammatory markers even if glucose levels are higher [46,47]. Importantly, overtreatment of hypoglycemia often results in rebound hyperglycemia. This glucose variability might induce an additional inflammatory stimulus such that rebound hyperglycemia might have a greater effect in impairing endothelial function and activating thrombosis than hypoglycemia alone [50]. On the other hand, recovery of hypoglycemia with normoglycaemia is accompanied by improvement in endothelial dysfunction and inflammation [51]. However, reactive hyperglycemia, following recovery from hypoglycemia, results in the deterioration of all these parameters.

Hypoglycemia can impair the release of vasoactive substances. The endothelium is responsible for the release of several substances, the most important being nitric oxide and endothelin-1 [52]. Following hypoglycemia in patients with T1D, the plasma levels of endothelin-1, the most potent vasoconstrictor, increase by 70% [53]. Impaired nitric oxide-mediated vasodilation is evident during hypoglycemia [47].

### 3.5. Oxidative Stress

Oxidative stress plays an important role in tissue damage caused by hypoglycemia. Several studies have shown an association between hypoglycemia and reactive oxygen species (ROS) production. In vitro studies in human umbilical vein endothelial cells showed that glucose deprivation stimulates mitochondrial ROS generation [54]. Insulin-induced recurrent hypoglycemia in streptozotocin-induced diabetic rats leads to an increase in malondialdehyde levels and a reduction in aconitase activity, which are indicators of oxidative stress in brain mitochondria [55]. Another study investigated the effects of hypoglycemia on the mitochondrial antioxidant system and the fragmentation of nuclear DNA in different brain regions in rats [56]. Following hypoglycemia, a decrease in the activity of mitochondrial antioxidant enzymes was observed that might be attributed to ROS generation, which in turn activates DNA damage. In healthy subjects, insulin-induced hypoglycemia is associated with an increase in lipid peroxidation markers and ROS production [45]. Exposure of insulin-treated streptozotocin-diabetic rats to recurrent hypoglycemia leads to metabolomic alterations in the hippocampus, as well as increased cerebral ischemic damage potentially via enhanced mitochondrial dysfunction [57,58].

### 3.6. Alterations of Brain Glucose Metabolism

Glucose is a critical energy substrate required for brain functioning. Various functional and metabolic neuroimaging techniques have been employed and have shown that hypoglycemia modulates multiple metabolic pathways in the brain [59]. Moderate hypoglycemia does not affect glucose uptake into the brain, and cerebral glucose metabolism is preserved, at least globally. However, at the regional level, moderate hypoglycemia causes a redistribution of cerebral blood flow to brain areas involved in the detection of hypoglycemia, particularly the hypothalamus. Recurrent hypoglycemia induces cerebral adaptive changes in glucose counterregulatory mechanisms at many different levels, including activation or deactivation of brain areas involved in behavioral responses, change in regional blood flow, as well as consumption of sources of energy other than glucose, particularly lactate. There is conflicting evidence as to whether recurrent hypoglycemia can stimulate brain glucose uptake, with most neuroimaging studies finding no evidence.

Overall, the hypoglycemia-induced changes in blood coagulability, inflammatory response, cell adhesion, release of vasoactive substances, and oxidative stress promote the development of endothelial dysfunction, which is an early marker for atherosclerosis [60,61]. Furthermore, hypoglycemia activates the renin-angiotensin-aldosterone system and increases aldosterone, which activates the mineralocorticoid receptor, further exacerbating endothelial dysfunction [62]. Promotion of atherogenesis due to exposure to recurrent episodes of severe hypoglycemia has been suggested as a potential mechanism in patients with T1D and IAH, in whom flow-mediated brachial dilatation was lower and femoral intima–media thickness was higher compared to patients with T1D without a history of severe hypoglycemia [63].

## 4. Data from Clinical Trials and Real-World Studies

In addition to the evidence from experimental studies, several clinical trials and real-world studies suggest an association between hypoglycemia and cardiovascular events in patients with diabetes.

### 4.1. Clinical Trials Exploring the Effects of Intensive Glucose-Lowering Therapy

Several large-scale clinical trials examined the effects of intensive versus conventional glucose-lowering therapy on cardiovascular outcomes in patients with diabetes (Table 2). The DCCT/EDIC study demonstrated the benefits of intensified insulin treatment and strict glycaemic control in T1D patients with a disease duration of 1–15 years, with regards to limiting the risk of vascular complications after a follow-up period of more than 12 years [64,65]. However, these benefits were associated with a threefold higher rate of severe hypoglycemia. Although the UKPDS study in newly diagnosed adults with T2D found no significant reduction in cardiovascular complications, a 10-year follow-up study showed a modest but statistically significant reduction of cardiovascular risk [66,67]. Higher rates of severe hypoglycemia were observed with intensive treatment. The ACCORD study, in T2D patients with established cardiovascular disease or multiple relevant risk factors, was interrupted early due to increased overall and cardiovascular mortality in the intensive treatment arm [68]. Although this study demonstrated increased hypoglycemia risk in the intensive treatment group, further analysis concluded that hypoglycemia did not appear to account for the difference in mortality between the two groups [69,70]. The ADVANCE study, in patients with T2D and vascular complications or at least one cardiovascular risk factor, did not reveal a reduction in cardiovascular risk with intensive glucose-lowering therapy after the 6-year post-trial follow-up [71,72]. However, severe hypoglycemia was more common in the intensive treatment group. Similarly, the VADT study in military veterans with poorly controlled T2D showed an increased hypoglycemia rate with intensive therapy but again without cardiovascular benefit [73]. The ORIGIN study in patients with cardiovascular risk factors plus impaired fasting glucose, impaired glucose tolerance, or T2D also showed that intensive glucose-lowering therapy was associated with increased severe hypoglycemia risk, but the effect on cardiovascular outcomes was the same with the conventional therapy [74]. 

### 4.2. Studies with Concomitant CGM and ECG Monitoring

Several studies have been performed in patients with T1D and T2D using concomitantly CGM and electrocardiogram (ECG) monitoring in an ambulatory setting. In patients with insulin-treated T2D and established cardiovascular disease or cardiovascular risk factors undergoing 5 days of concomitant CGM and ECG monitoring, the incidence of bradycardia was eightfold higher, and the incidence of atrial and ventricular ectopic beats was fourfold higher during nocturnal hypoglycemia compared with nocturnal normoglycaemia [38]. During the day, a small but significant increase in ventricular premature beats was observed, whereas no episodes of bradycardia were detected. Simultaneous CGM and ECG monitoring for 4 days in T1D patients revealed that asymptomatic hypoglycemia was common (76% of nocturnal and 39% of daytime hypoglycemia) and that differences existed in cardiac arrhythmias during day and night [75]. Specifically, bradycardia was more frequent during nocturnal hypoglycemia compared with nocturnal normoglycaemia. Bradycardia was less frequent, and atrial ectopics were more frequent during daytime hypoglycemia compared with nighttime hypoglycemia. In another study, ventricular arrhythmias were observed in association with asymptomatic hypoglycemia during simultaneous CGM and ECG recording for 5 days in patients with T2D and documented cardiovascular disease treated with insulin and/or sulfonylureas, particularly during the night [76]. Furthermore, concomitant CGM and ECG monitoring for 3 days in insulin-treated T2D patients with coronary heart disease showed that chest pain was accompanied by ischemic changes in the ECG of some of the patients during hypoglycemia [77].

### 4.3. ‘Dead in Bed’ Syndrome

An association has been suggested between hypoglycemia and the unexpected nocturnal death of patients with T1D without cardiovascular disease, described as ‘dead in bed’ syndrome. A retrospective analysis of 50 sudden overnight deaths in patients with T1D was carried out, and in 22 cases, no cause was found [78]. All patients were young (12–43 years) with a history of nocturnal hypoglycemia. The ‘dead in bed’ syndrome is thought to be caused by a hypoglycemia-induced fatal arrhythmia during sleep. Since then, it has been reported in a series of studies, with one of them calculating a tenfold increase in sudden death in young patients with T1D compared with the nondiabetic population [79]. Importantly, although the ‘dead in bed’ syndrome has been described in T1D, hypoglycemia-induced fatal arrhythmias are also likely to occur in patients with insulin-treated T2D.

### 4.4. Studies on Cerebral Ischemia and Stroke

Hypoglycemia can cause localized cerebral neuroglycopenia and transient focal neurological deficits, usually resolving after correction of the low glucose levels. Case-report neuroimaging studies have shown areas consistent with ischemia in the case of a neurological deficit following severe hypoglycemia [80,81]. The data linking hypoglycemia with stroke risk are limited. A report from the ORIGIN trial showed that severe hypoglycemia was associated with increased risk of the composite outcome of non-fatal myocardial infarction, stroke or cardiovascular death; hazard ratio 1.58, 95% CI 1.24–2.02 [82]. In another study, patients with ischemic stroke received intensive insulin therapy or subcutaneous insulin for 24 h [83]. It was found that in the intensive insulin therapy group, glucose values <126 mg/dL were more frequent (95.4% versus 67.4%, *p*-value < 0.0001) and infarct growth was greater (27.9 cm versus 10.8 cm, *p*-value = 0.04). However, after 3 months, functional outcomes, serious adverse events and death rates were similar between the two groups. Within 24 h of ischemic stroke, glucose potassium insulin (GKI) infusion compared to saline infusion was associated with a higher incidence of asymptomatic hypoglycemia [84]. In a secondary analysis, GKI was associated with greater infarct growth in patients with complete intracranial vessel occlusion. Additionally, in patients with subarachnoid hemorrhage, glucose <80 mg/dL was associated with vasospasm, cerebral infarction, and functional disability at 3 months [85]. Another study found no association between severe hypoglycemia and the risk of stroke [86].

### 4.5. Studies on Peripheral Artery Disease

Limited data are available with regard to the impact of hypoglycemia on peripheral artery disease (PAD). Among 6713 patients with T2D admitted to the hospital, 80 had severe hypoglycemia in the preceding three months, and 304 had symptomatic hypoglycemia in the last month [87]. It was demonstrated that both symptomatic and severe hypoglycemia was associated with increased risk for cardiovascular disease, defined as coronary heart disease, stroke or PAD; the odds ratio, 95% CI, were 2.64, 1.85–3.76 and 6.59, 3.79–11.45, respectively. Additionally, analysis of the prospective DiaRegis registry showed that PAD prevalence was higher in patients with T2D who had experienced hypoglycemia during the 12-year follow-up compared to those without hypoglycemia; 8.6% versus 5.7%, *p*-value < 0.05 [88]. Importantly, hypoglycemia is an independent predictor of amputations in patients hospitalized for acute diabetic foot [89].

### 4.6. Meta-Research Data

A systematic review and meta-analysis were conducted to explore the association between hypoglycemia and increased cardiac arrhythmia risk in adults with T1D or T2D, comparing ECG changes during episodes of hypoglycemia and normoglycaemia [90,91,92]. Hypoglycemia was associated with a reduction in most components of heart rate variability and an increase in the prevalence of arrhythmias (bradycardia, ventricular premature beats, atrial premature beats, atrial ectopic beats). The meta-analysis of 15 studies showed that the QT interval was more significantly prolonged during hypoglycemia compared to normoglycaemia. Another meta-analysis assessed the association between hypoglycemia and vascular events in older subjects (mean age >60 years) who had pre-diabetes or diabetes [90]. The meta-analysis of 8 studies demonstrated that hypoglycemia was associated with macrovascular complications (odds ratio, OR 1.83, 95% CI 1.64–2.05) and increased likelihood of overall mortality (OR 2.04, 95% CI 1.68–2.47). Another systematic review and meta-analysis that included ten studies found that severe hypoglycemia was associated with increased cardiovascular risk (relative risk 1.91, 95% CI 1.69–2.15) [91]. Additionally, a meta-analysis of the ACCORD, ADVANCE, UKPDS and VADT trials showed that patients receiving intensive glucose control treatment had significantly more major hypoglycaemic events (hazard ratio 2.48, 95% CI 1.91–3.21), but there was no significant effect on the risk of stroke [93].

## 5. Conclusions

There is extensive evidence suggesting that hypoglycemia is associated with significant cardiovascular effects in patients with diabetes. Based on data from experimental studies, a number of pathophysiological mechanisms have been proposed, linking hypoglycemia to increased cardiovascular risk. These mechanisms include hemodynamic changes, myocardial ischemia, abnormal cardiac repolarization, cardiac arrhythmias, procoagulant and prothrombotic effects, proinflammatory effects, and induction of oxidative stress, promoting the development of endothelial dysfunction and atherogenesis. Data from large-scale clinical trials and real-world studies, as well as meta-research evidence, confirm the proposed association between hypoglycemia and adverse cardiovascular events in patients with diabetes. Limited data are available with regard to the impact of hypoglycemia on stroke risk and peripheral artery disease.

Whether the association between hypoglycemia and increased cardiovascular risk is causal or due to confounding factors remains uncertain. Hypoglycemia can probably trigger cardiovascular events in vulnerable patients who are at high cardiovascular risk and may also interact with other risk factors. It is difficult to confirm causality due to the observational nature of many studies and the inability to capture all hypoglycemic episodes, which may be mild or asymptomatic. New drugs, such as sodium-glucose co-transporter inhibitors and glucagon-like peptide-1 receptor agonists that do not cause hypoglycemia and are associated with cardiovascular benefits, are available for the management of T2D. The use of new technologies, including CGM devices and insulin pumps, is beneficial in reducing hypoglycemia in T1D. It is also important for clinicians to recognize patients at high risk of hypoglycemia, IAH and those at increased cardiovascular risk during hypoglycemia. In any case, appropriate and individualized glycemic goals and continuous education of patients with diabetes on dietary modification, exercise management, glucose monitoring and medication adjustment are needed to effectively prevent, recognize and treat hypoglycemia.

## Figures and Tables

**Table 1:** Glucose thresholds for hormonal counter-regulation and clinical features of hypoglycemia.

Blood Glucose Thresholds *	Hormone Responses
<80–85 mg/dL	↓ Insulin
<65–70 mg/dL	↑ Glucagon
<65–70 mg/dL	↑ Epinephrine
<65–70 mg/dL	↑ Cortisol and growth hormone
Symptoms and signs (usually when glucose < 50–58 mg/dL)	
Autonomic:	
Adrenergic: tremors, palpitations/tachycardia, pallor, anxiety, and arousal.	
Cholinergic: sweating, hunger, salivation, nausea, and paraesthesias.	
Neuroglycopenic:	
Weakness, fatigue, confusion, behavioral changes, emotional lability, slurred speech, blurred vision, headache, dizziness, seizures, incoordination, focal neurological deficits (diplopia, hemiparesis), and loss of consciousness. If hypoglycemia is severe and prolonged, brain damage and even death may occur.	

* Glycaemic thresholds for activation of hormonal counter-regulation and awareness of hypoglycemia may be lower in some people with T1D, especially in those with frequent hypoglycemia, long disease duration or autonomic neuropathy. Please note that glucose values refer to arterialized venous glucose concentration. Notes: ↑: increase; ↓: decrease.

**Table 2:** Large-scale clinical trials exploring the effects of intensive versus conventional glucose-lowering therapy.

Study	Patients	CV Outcomes with Intensive Therapy	Hypoglycemia Risk with Intensive Therapy
DCCT/EDIC [64,65]	T1D adolescents and adults	↓ CVD only evident in the follow-up trial after >12-yr.	↑ severe hypoglycemia
UKPDS [66,67]	Newly diagnosed T2D adults	↓ CVD only evident in the follow-up trial after >10-yr.	↑ severe hypoglycemia
ACCORD [68]	T2D adults with CVD or CV risk factors	Early interrupted due to ↑ overall and CV mortality	↑ severe hypoglycemia
ADVANCE [71]	T2D adults with vascular complications or CV risk factors	No CV benefit	↑ severe hypoglycemia
VADT [73]	T2D adults poorly controlled	No CV benefit	↑ hypoglycemia
ORIGIN [74]	Adults with CV risk factors plus prediabetes or T2D	No CV benefit	↑ severe hypoglycemia

Abbreviations: CV: cardiovascular; CVD: cardiovascular disease; T1D: type 1 diabetes; T2D: type 2 diabetes. Notes: ↑: increase; ↓: decrease.

## Data Availability

All data generated or analyzed during this study are included in this published article. Anonymized data will be shared by request from any qualified investigator.

## Abbreviations

aPTT: activated partial thromboplastin time; CD40: cluster of differentiation 40; CGM: continuous glucose monitoring; CI: confidence interval; CRP: C-reactive protein; CV: cardiovascular; CVD: cardiovascular disease; DBP: diastolic blood pressure; ECG: electrocardiogram; EF: ejection fraction; GKI: glucose potassium insulin; HAAF: hypoglycemia associated autonomic failure; HR: heart rate; IAH: impaired awareness of hypoglycemia; ICAM: intercellular adhesion molecule; IL: interleukin; OR: odds ratio; PAD: peripheral artery disease; PAI: plasminogen activator inhibitor; ROS: reactive oxygen species; SBP: systolic blood pressure; sCD40L: soluble cluster of differentiation 40 ligand; T1D: type 1 diabetes; T2D: type 2 diabetes; TNFα: tumor necrosis factor-alpha; tPA: tissue plasminogen activator; VCAM: vascular cell adhesion molecule; VEGF: vascular endothelial growth factor; vWF: von Willebrand factor.
